# Dissociative identity state-dependent working memory in dissociative identity disorder: a controlled functional magnetic resonance imaging study

**DOI:** 10.1192/bjo.2022.22

**Published:** 2022-04-11

**Authors:** Eline M. Vissia, Andrew J. Lawrence, Sima Chalavi, Mechteld E. Giesen, Nel Draijer, Ellert R. S. Nijenhuis, André Aleman, Dick J. Veltman, Antje A. T. S. Reinders

**Affiliations:** Cognitive Neuroscience Centre, University Medical Centre Groningen, University of Groningen, The Netherlands; and Centre for Psychotrauma, Heelzorg, The Netherlands; Department of Psychological Medicine, Institute of Psychiatry, Psychology & Neuroscience, King's College London, UK; Cognitive Neuroscience Centre, University Medical Centre Groningen, University of Groningen, The Netherlands; and Research Centre for Movement Control and Neuroplasticity, Department of Movement Sciences, Katholieke Universiteit Leuven, Belgium; Cognitive Neuroscience Centre, University Medical Centre Groningen, University of Groningen, The Netherlands; Department of Psychiatry, VU University Medical Center, Amsterdam University Medical Center, The Netherlands; Clienia Littenheid AG, Switzerland; Cognitive Neuroscience Centre, University Medical Centre Groningen, University of Groningen, The Netherlands; Department of Psychiatry, VU University Medical Center, Amsterdam University Medical Center, The Netherlands; Cognitive Neuroscience Centre, University Medical Centre Groningen, University of Groningen, The Netherlands; and Department of Psychological Medicine, Institute of Psychiatry, Psychology & Neuroscience, King's College London, UK

**Keywords:** dissociative disorders, simulation, post-traumatic stress disorder, trauma, cognitive neuroscience

## Abstract

**Background:**

Memory function is at the core of the psychopathology of dissociative identity disorder (DID), but little is known about its psychobiological correlates.

**Aims:**

This study aims to investigate whether memory function in DID differs between dissociative identity states

**Method:**

Behavioural data and neural activation patterns were assessed in 92 sessions during an *n*-back working memory task. Participants were people with genuine diagnosed DID (*n* = 14), DID-simulating controls (*n* = 16) and a paired control group (post-traumatic stress disorder (*n* = 16), healthy controls (*n* = 16)). Both DID groups participated as authentic or simulated neutral and trauma-related identity states. Reaction times and errors of omission were analysed with repeated measures ANOVA. Working memory neural activation (main working memory and linear load) was investigated for effects of identity state, participant group and their interaction.

**Results:**

Identity state-dependent behavioural performance and neural activation was found. DID simulators made fewer errors of omission than those with genuine DID. Regarding the prefrontal parietal network, main working memory in the left frontal pole and ventrolateral prefrontal cortex (Brodmann area 44) was activated in all three simulated neutral states, and in trauma-related identity states of DID simulators, but not those with genuine DID or post-traumatic stress disorder; for linear load, trauma-related identity states of those with genuine DID did not engage the parietal regions.

**Conclusions:**

Behavioural performance and neural activation patterns related to working memory in DID are dependent on the dissociative identities involved. The narrowed consciousness of trauma-related identity states, with a proneness to re-experiencing traumatising events, may relate to poorer working memory functioning.

Working memory has been defined as a limited capacity system for the temporary maintenance and manipulation of information necessary to execute complex tasks.^1^ Stress, and especially chronic stress, may have a major effect on the working memory system;^2,3^ for example, neuropsychological studies have shown various neurocognitive deficits in post-traumatic stress disorder (PTSD), including in working memory (for a quantitative meta-analysis, see Scott et al^4^). Major stress may negatively affect prefrontal cortex operations,^2^ and several brain imaging studies have demonstrated working memory deficits in PTSD that are associated with altered prefrontal activation.^4^ However, little is known about working memory functioning and its neural correlates in severely traumatised samples suffering from dissociative pathology, with dissociative identity disorder (DID) being considered the most severe of trauma-related psychiatric disorders.^5^ The most replicated difference between DID and simulating controls is a deficit in cognitive processing (in DID) for memory and reaction times in general,^6,7^ but identity state-dependent neural correlates of these differences remain unexplored. It is therefore important to investigate identity state-dependent working memory functioning in DID compared with working memory functioning in individuals with PTSD and DID-simulating controls.

One functional magnetic resonance imaging (fMRI) study investigated the neural correlates of working memory performance in patients with a dissociative disorder and showed, in comparison with healthy controls,^8^ that the patients had enhanced working memory performance and increased activation in the prefrontal parietal network (PPN^9,10^). However, the data in this study were obtained in a single, undefined identity state. Although it is likely that the data were acquired in the most prominent neutral identity state, it remains unclear whether the reported brain activations are dissociative identity state-dependent, whether these results can be simulated by DID-simulating controls and whether similar brain activation patterns are present in PTSD. Therefore, the current study aimed to investigate behavioural and neural correlates of working memory in both a neutral and trauma-related identity state in genuine DID, and to directly compare these with a DID-simulating control group and a control group consisting of matched pairs of healthy controls and individuals with PTSD. The commonly used *n*-back test was chosen to assess working memory. We hypothesise that (a) working memory task-dependent behavioural performance and neural activation in DID is identity state-dependent, with a neutral identity state showing better behavioural task performance and neural activation in the PPN when compared with a trauma-related identity state; (b) identity state-dependent differences in genuine DID are similar to those that distinguish healthy controls and PTSD, namely that a trauma-related identity state and PTSD show similarly impaired working memory and, based on the above-mentioned fMRI study, a neutral identity state would show equal or enhanced working memory performance and increased neural PPN activation compared with healthy controls; (c) differences in behavioural performance and neural activation patterns are expected between patients with genuine DID and DID-simulating controls, who are hypothesised to behave comparably to healthy controls.

## Method

### Participants

According to the DSM-5,^11^ DID is characterised by the experience of two or more dissociative identity states. Following previously used terminology^12^ and conceptualisation,^13^ two prototypes of dissociative identity states can be described: neutral identity state (NIS) and trauma-related identity state (TIS). NIS is considered a hypoaroused identity state with overmodulation of emotion^14^ and mental avoidance of trauma-related cues.^12,13,15^ In contrast, TIS is usually, but not always,^16^ a hyperaroused identity state, with undermodulation of emotion^14^ and prominent emotional and somatic reactions to trauma-related cues.

Sixty-two individuals participated in the *n*-back study: 14 people with genuine diagnosed DID (DID-G), 16 DID-simulating healthy controls (DID-S), and a paired control group of 16 individuals with PTSD and 16 healthy controls. Participants in the latter two groups were individually paired on the basis of age and education, to present a control group consisting of a TIS and a NIS; these control group members were tested as themselves. All groups were matched for age, level of education and Western European ancestry. All participants were female because only women with DID volunteered to take part in this study. This study was part of a larger multicentre study investigating the neurobiology of DID.^17–19^ The working memory-related data and results have not been published in a peer-reviewed journal, but the sample characteristics, simulation instructions/paradigm and the inclusion and exclusion criteria have been described in detail before,^19^ as well as comorbid disorders.^20,21^ Therefore, only a brief summary is provided in Supplementary Appendix 1 and Table 1 available at https://doi.org/10.1192/bjo.2022.22.^20^

### Procedure

After enrolment, participants received a complete description of the study and gave written informed consent according to procedures approved by the Medical Ethical Committee of the University Medical Center Groningen (reference number: METC2008.211) and Amsterdam Medical Center (reference number: MEC09/155). Three assessment sessions were completed. During the first session, participants completed questionnaires assessing dissociative symptoms, trait anxiety and potentially traumatising events (the Dissociative Experiences Scale,^22^ Somatoform Dissociation Questionnaire-20,^23^ State-Trait Anxiety Inventory and Traumatic Experiences Checklist^24^). The DID-G group were asked to fill in these questionnaires as their prominent NIS, a dissociative identity state that primarily fulfils important tasks in daily life, and the other participants filled out the questionnaires as their normal selves. The DID-S group completed these measures as themselves. Together with their therapists, the DID-G group decided which NIS and TIS they would be willing and able to participate as (descriptions of these states have been previously provided^19^). Researchers N.D. and E.R.S.N. confirmed the suitability of the two identity states: those participating as the TIS had to be able to recollect traumatic memories and possess a tendency toward hyperarousal in reaction to cues they regarded as threatening,^16^ whereas those participating as the NIS had to strive to mentally avoid these memories.^12,15,25^ The ability to read was another requirement for both identity states.

In the second session, all participants were given the opportunity to experience the fMRI environment with a practice session of the *n*-back task in a dummy scanner, to reduce anticipation anxiety (see Supplementary Appendix 1 for a detailed description). The final session was the fMRI scanning session. Immediately before going into the scanner, all participants completed a full run practice session of the *n*-back task. DID-G and DID-S groups completed the fMRI procedure in both the selected NIS and TIS, and PTSD and healthy controls completed it once as themselves. Depending on the DID-G group's preference, participation of both identity states occurred on one day or on two separate days. The presence of the selected identity state was confirmed immediately before and after the *n*-back task, by means of a short verbal inquiry (M.E.G. or E.M.V.). The order in which identity states participated was similar for the two groups (DID-G: TIS started in six of 14 participants; DID-S: TIS started in six of 16 participants).

### Statistical analyses

#### Behavioural performance

Behavioural data acquired when participants were in the scanner were extracted, summarised and analysed with R software (version 3.6.1 for Windows, R Foundation for Statistical Computing, Vienna, Austria; see https://www.R-project.org/), with repeated measures ANOVA conducted using the Analysis of Factorial Experiments package (afex;^26^ version 0.25.1; https://CRAN.R-project.org/package=afex). An intrinsic match exists in the DID-G and DID-S groups between identity states (NIS and TIS), and controls (PTSD and healthy controls) were matched for age, gender and education. This enabled us to include dissociative identity state as a within-group factor to achieve an optimal comparison between the groups.

Two measures of behavioural performance during the scanning task were analysed in terms of task difficulty. These were average reaction time (seconds) calculated over correct responses, and the proportion of trials with errors of omission (i.e. *n*-back targets that were not responded to). These were calculated separately for each task condition, and performance was analysed with three-way factorial repeated measures ANOVA. Identity state (NIS or TIS) and task condition (zero-back, one-back, two-back or three-back) were within-participant factors, and group (DID-G, DID-S or control) was a between-participants factor. A variance-stabilising arcsine inverse square-root transform was applied to the proportion data for analysis. Results are reported with Greenhouse–Geisser adjustments for non-sphericity. *P*-values <0.05 are reported as significant findings and *P*-values between 0.05 and <0.1 are reported as trends, to take into consideration the relatively low number of patients included in this study.

Task behavioural data from the scanning session was missing for one individual in the DID-G group because of a technical error with the response logging equipment. This individual was included for functional analysis after careful inspection confirmed that they were not an outlier for behavioural data on the practice tasks, nor for the functional data in the Statistical Parametric Mapping analysis. Participants did not receive feedback on their task responses, so there would be no practical difference in the experience of the task for this individual. In addition, for the behavioural analysis of reaction time, two further individuals in the DID-G group could not be included in the repeated measures ANOVA because they did not respond to any stimuli in one of the task conditions (three-back condition, TIS state) within the trial time limit.

#### Neuroimaging data

For acquisition parameters and preprocessing details, see Supplementary Appendix 1. fMRI data were analysed with SPM12 for UNIX (Wellcome Trust Centre for Neuroimaging; http://www.fil.ion.ucl.ac.uk). The general linear model was used for statistical analyses. Individual participant data was modelled with a block design, using boxcar regressors convolved with a canonical haemodynamic response function. Next, contrast images containing parameter estimates for the main effect of task (task versus baseline) and task load (based on Mannie et al^27^) were entered into second-level random effects analyses. The main effect of task assesses main working memory (MWM) and compares the effect of the task to baseline independent of task difficulty, i.e. one-, two- and three-back versus zero-back. Task load considers activation that changes linearly with task difficulty from zero-back to three-back, termed the linear load of the task. Two sets of comparisons are made for both MWM and linear load: within-group comparisons and between-group comparisons. For the within-group comparisons, the main effect of task was assessed within each group and each identity state for both MWM and linear load. Interaction effects between NIS and TIS were calculated within group as well. Between-group comparisons were made for the groups for each identity state and for MWM and linear load. Finally, we tested the NIS and TIS interaction effects between groups for both MWM and linear load.

The initial significance level was set at *P* < 0.05, and was family-wise error multiple comparisons corrected for the whole brain. Brain regions surviving this stringent threshold were reported. Additionally, we created a mask (see Supplementary Appendix 1) to be able to apply a less stringent multiple comparison correction, given this smaller volume of specific interest. Areas reaching significance at *P* < 0.05 after being family-wise error-corrected for multiple comparisons for the small volume defined by the mask were reported. We also reported brain areas within the mask at an explorative threshold of *P* < 0.005 uncorrected, in combination with an extent voxel threshold of more than nine, to reduce the risk of type 1 error.^28^ This threshold was chosen to account for the spatial resolution of the data.

## Results

### Reaction times and omission errors

**Fig. 1:**
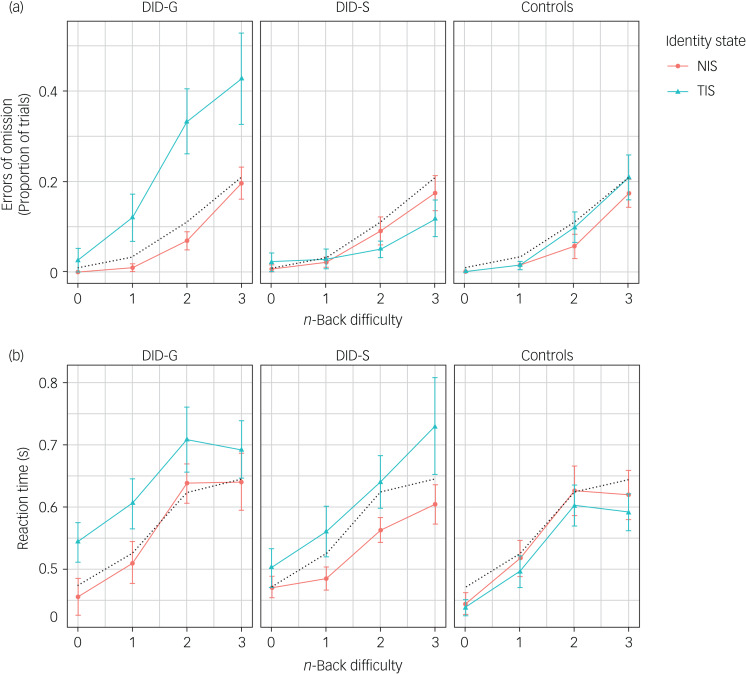
*n*-Back behavioural results. (a) Group average proportion of omission errors made for the *n*-back working memory task during the fMRI scan session. Results show zero-, one-, two- and three-back (*x*-axis) for the NIS and TIS of DID-G and DID-S participants, and for post-traumatic stress disorder and healthy controls as control groups. Error bars display ±2 s.e.m. The dotted grey line shows the average proportion of omission errors for a given task condition averaged over all participants and identity states. This reference line is the same in all panels. (b) Group average mean reaction time results (in seconds) for the *n*-back working memory task during the fMRI scan session. Results show zero-, one-, two- and three-back (*x*-axis) for the NIS and TIS of DID-G and DID-S participants, and for PTSD and healthy controls as control groups. Values are mean average with error bars displaying ±2 s.e.m. The dotted grey line shows the average reaction time for a given task condition averaged over all participants and identity states. This reference line is the same in all panels. There is evidence of an effect of identity state for errors of omission, such that errors of omission were increased in the TIS in the DID-G group. The reaction time data showed an effect of identity state on task performance in the DID-G and DID-S groups, such that responses were slowed in the authentic and simulated TIS relative to the authentic and simulated NIS, for all working memory loads, i.e. task difficulty. Reaction times in DID-G and DID-S groups were slowed by ~75 ms on average for TIS. In contrast, patients with PTSD did not show slowed reaction times relative to matched healthy controls. DID-G, genuine diagnosed dissociative identity disorder; DID-S, dissociative identity disorder-simulating healthy controls; fMRI, functional magnetic resonance imaging; NIS, neutral identity state; PTSD, post-traumatic stress disorder; TIS trauma-related identity state.

Behavioural data from the scanner task are presented in Fig. 1(a) and 1(b), which show errors of omission and reaction time, respectively. Means and s.d. are presented in Table 1.

**Table 1:** Working memory behavioural performance

	Group	Identity state	*n*-Back condition				Task average
			0-back	1-back	2-back	3-back	
Reaction times (s)	DID-G (*n* = 11)	NIS	0.456 (0.098)	0.51 (0.111)	0.638 (0.105)	0.641 (0.152)	0.555 (0.104)
		TIS	0.543 (0.105)	0.605 (0.134)	0.708 (0.175)	0.692 (0.153)	0.621 (0.106)
	DID-S (*n* = 16)	NIS	0.471 (0.067)	0.485 (0.075)	0.563 (0.08)	0.604 (0.127)	0.527 (0.07)
		TIS	0.502 (0.125)	0.56 (0.163)	0.64 (0.171)	0.73 (0.31)	0.602 (0.18)
	Controls (*n* = 16)	NIS	0.444 (0.068)	0.517 (0.116)	0.626 (0.16)	0.619 (0.157)	0.546 (0.105)
		TIS	0.438 (0.051)	0.496 (0.103)	0.602 (0.132)	0.592 (0.118)	0.524 (0.074)
Omission proportion	DID-G (*n* = 13)	NIS	0 (0)	0.01 (0.03)	0.07 (0.07)	0.2 (0.13)	0.07 (0.04)
		TIS	0.03 (0.09)	0.12 (0.19)	0.33 (0.26)	0.43 (0.36)	0.23 (0.2)
	DID-S (*n* = 16)	NIS	0.01 (0.03)	0.02 (0.04)	0.09 (0.12)	0.17 (0.16)	0.07 (0.06)
		TIS	0.02 (0.08)	0.03 (0.09)	0.05 (0.07)	0.12 (0.16)	0.05 (0.06)
	Control (*n* = 16)	NIS	0 (0)	0.01 (0.04)	0.06 (0.11)	0.17 (0.12)	0.06 (0.05)
		TIS	0 (0)	0.01 (0.04)	0.1 (0.13)	0.21 (0.2)	0.08 (0.06)

Working memory behavioural performance: cell means and s.d. are presented for reaction time (correct responses only) and proportion of responses omitted (nine responses per condition). DID-G, diagnosed genuine dissociative identity disorder; NIS, neutral identity state; TIS, trauma-related identity state; DID-S, simulated dissociative identity disorder.

First, as expected, the experimental manipulation of task difficulty had a significant effect on both errors of omission (main effect: F_(2.62, 109.96)_ = 100.10, *P* < 0.0001) and reaction times (main effect: F_(2.23, 89.36)_ = 63.2, *P* < 0.0001), regardless of group or identity state. This was such that reaction times were slower and more errors of omission were made as the task difficulty increased. *Post hoc* tests for linear trend confirmed that this effect was significant in all groups and identity states (F-range: 6.54–32.3, *P*-range: 0.001 to <0.0001), with the single exception of errors of omission within the DID-S TIS cell, which approached significance (F_(1.93, 28.91)_ = 3.23, *P* = 0.06).

For errors of omission, participating groups had statistically significant interactions with both identity state (F_(1, 42)_ = 4.64, *P* = 0.04) and task condition (F_(5.24, 109.96)_ = 3.77, *P* = 0.003), and the omnibus group×identity state×task condition interaction approached significance (F_(4.50, 94.51)_ = 2.10, *P* = 0.08). Exploration of these effects employing *post hoc* within-participant group ANOVAs revealed significant effects of identity state only in the DID-G group (F_(1, 12)_ = 6.88, *P* = 0.02), along with a significant identity state×task condition interaction (F_(1.50, 18.02)_ = 4.97, *P* = 0.03). In other groups, these effects did not reach significance (F-range: 0.43–1.43, *P*-range: 0.59–0.25). A complementary analysis comprising a *post hoc* ANOVA conducted within each identity state demonstrated that the main effect of group and the group×task condition interaction were only significant for the TIS (F_(2, 42)_ = 8.43, *P* = 0.0008, and F_(4.61, 96.72)_ = 4.07, *P* = 0.003, respectively). In contrast, these same effects were non-significant for the NIS (F_(2, 42)_ = 0.48, *P* = 0.62, and F_(4.98, 104.58)_ = 0.60, *P* = 0.70, respectively).

Reaction times were additionally affected by identity state (main effect: F_(1, 40)_ = 4.45, *P* = 0.041), interpretable as slower reactions in the TIS (estimated marginal mean difference: +44.5 ms). However, this occurred in the presence of a trend-level group×identity interaction (F_(2, 40)_ = 2.52, *P* = 0.09), visible in Fig. 1(b) as the greater (and opposite direction) effects of identity state in the DID-G (+75.9 ms, 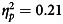) and DID-S (+77.3 ms, 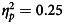) groups compared with controls (−19.7 ms, 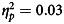). There was no significant main effect of group (F_(2, 40)_ = 1.17, *P* = 0.32), and no significant group×condition interaction (F_(4.47, 89.36)_ = 1.20, *P* = 0.32). There was no identity×condition interaction (F_(2.56, 102.38)_ = 0.17, *P* = 0.89). The omnibus interaction (group×identity×condition) was also non-significant (F_(5.12, 102.38)_ = 1.36, *P* = 0.25).

### Imaging data

#### Neural correlates of MWM

##### Within group: main effects of task

**Fig. 2:**
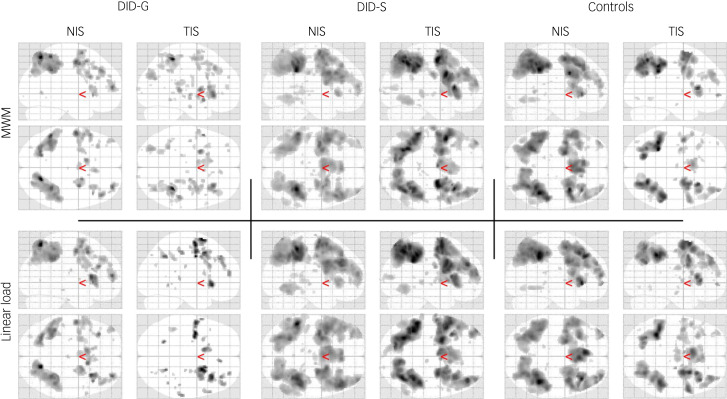
Neural correlates of working memory. Glass brain presentation for the *n*-back main effects per group and dissociative identity state for MWM (top) and linear load (bottom) at 0.005 uncorrected. Sagittal and axial views for both NIS and TIS of DID-G are displayed on the left side, DID-S are displayed in the middle, and controls are displayed on the right side where the NIS are the normal controls and the TIS are the patients with PTSD. The DID-G TIS shows less activation in the prefrontal parietal network as compared with the DID-G NIS, and the NIS and TIS of the controls and DID-S group. The DID-S TIS shows most activation in the prefrontal parietal network. Red arrows represent the 0,0,0 coordinate. DID-G, genuine diagnosed dissociative identity disorder; DID-S, dissociative identity disorder-simulating healthy controls; MWM, main working memory; NIS, neutral identity state; PTSD, post-traumatic stress disorder; TIS trauma-related identity state.

The main effect of MWM performance was associated with activation of the PPN in all groups. However, the extent to which areas activated in this brain network differed with group and identity state (see top parts of Fig. 2 and Table 2). Activation of the bilateral parietal cortex, right dorsolateral prefrontal cortex and right insula were found in all groups and identity states. The left frontal pole and left ventrolateral prefrontal cortex (VLPFC) were found for all three NIS and the TIS of the DID-S group, but not for the TIS of the DID-G group or the PTSD group.

**Table 2:** Group and identity state-dependent neural correlates of main working memory

				Main working memory		MNI				
			Right/left	Brain region	Brodmann area	*x*	*y*	*z*	*k*	*T*
Within group	Main effects of task	DID-G								
		NIS main effect								
			Right	Parietal cortex (superior)	7	18	−66	58	120	6.43*
			Right	Parietal cortex (inferior, angular)	39/40	36	−48	36	344	5.82*
			Left	Parietal cortex (inferior)	7/39	−40	−50	40	359	5.41**
			Right	Dorsolateral prefrontal cortex	9	46	36	36	69	4.99
			Left	Supplementary motor area	6	−10	10	50	37	4.84
			Left	Ventrolateral prefrontal cortex	44	−50	18	26	84	4.80
			Right	Insula	13	42	22	−4	134	4.24
			Left	Frontal pole	10	−40	48	18	30	3.75
			Right	Dorsolateral prefrontal cortex	9	36	32	26	10	3.33
		TIS main effect								
			Right	Parietal cortex (inferior)	7	36	−42	54	1	9.11***
			Right	Insula	13/44	32	26	−4	256	6.24*
			Right	Dorsolateral prefrontal cortex	9	40	28	38	78	4.90
			Left	Parietal cortex (inferior)	7	−32	−50	50	46	4.21
			Right	Parietal cortex (angular)	39	44	−50	36	15	4.13
			Left	Medial frontal gyrus (including anterior cingulate gyrus (dorsal part))	6/32	−8	10	48	71	3.84
		DID-S								
		NIS main effect								
			Left	Parietal cortex (inferior)	40	−52	−42	48	4	8.36***
			Right	Parietal cortex (inferior, superior, intra-parietal sulcus)	7/40	36	−44	36	331	11.67****
			Right	Dorsolateral prefrontal cortex	9	48	24	32	353	6.83*
			Left	Ventrolateral prefrontal cortex (including dorsolateral prefrontal cortex)	9/44/46	−42	12	28	395	6.52*
			Left	Parietal cortex (inferior)	7/40	−28	−56	42	467	5.98*
			Left	Medial frontal gyrus (including anterior cingulate gyrus (dorsal part))	6/32	−4	12	54	197	5.84*
			Right	Insula	13	38	24	−2	176	5.83*
			Left	Frontal pole (including dorsolateral prefrontal cortex)	10/46	−34	48	26	129	4.04
			Right	Parietal cortex (superior)	7	12	−76	52	125	3.93
			Left	Insula	45	−30	28	0	26	3.83
		TIS main effect								
			Right	Premotor cortex	6	28	−6	52	62	11.20***
			Left	Premotor cortex	6	−44	8	30	38	10.86***
			Left	Parietal cortex (inferior)	40	−52	−38	42	40	10.13***
			Right	Parietal cortex (angular)	39	40	−60	56	16	9.24***
			Left	Parietal cortex (inferior)	7/40	−36	−52	52	1	8.33***
			Left	Ventrolateral prefrontal cortex	44	−50	12	22	1	8.15***
			Left	Parietal cortex (inferior, superior)	7/40	−28	−56	46	450	10.73****
			Right	Insula	13/45	36	22	8	345	10.54****
			Right	Parietal cortex (inferior, superior, intra-parietal sulcus)	7/40	36	−46	38	386	9.20****
			Left	Ventrolateral prefrontal cortex (including dorsolateral prefrontal cortex)	6/44/46	−46	10	28	244	8.65*
			Right	Parietal cortex (superior) and praecuneus	7	10	−70	58	424	6.90*
			Right	Dorsolateral prefrontal cortex	9	42	40	30	297	6.48*
			Right	Medial frontal gyrus (including anterior cingulate gyrus (dorsal part))	8/32	6	14	46	226	4.83
			Left	Insula	13/45/47	−34	20	8	69	4.62
			Left	Frontal pole	10	−36	54	20	24	4.14
			Left	Frontal pole	10	−44	44	26	14	3.45
		Controls								
		Healthy controls main effect								
			Right	Premotor cortex	8	38	6	32	6	9.66***
			Right	Parietal cortex (superior)	7	34	−50	46	7	8.75***
			Left	Parietal cortex (superior)	7	−30	−58	50	3	8.35***
			Right	Parietal cortex (inferior)	40	52	−30	44	2	8.11***
			Left	Premotor cortex	6	−22	−2	54	1	8.04***
			Right	Parietal cortex (inferior, superior, intra-parietal sulcus)	7/40	44	−46	38	398	10.56****
			Left	Parietal cortex (inferior, superior, intra-parietal sulcus)	7/40	−28	−58	42	466	8.08****
			Right	Insula	13	38	26	−2	252	7.72*
			Left	Medial frontal gyrus (including anterior cingulate gyrus (dorsal part))	6/32	0	10	54	237	7.09*
			Left	Ventrolateral prefrontal cortex	44	−50	12	28	327	6.63*
			Right	Parietal cortex (superior) and praecuneus	7	6	−72	52	412	6.29*
			Right	Dorsolateral prefrontal cortex	9	46	32	30	226	5.67*
			Left	Insula	13/45	−32	22	0	142	5.61*
			Left	Frontal pole	10	−36	54	20	29	4.55
			Left	Putamen	49	−22	14	4	13	4.25
		PTSD main effect								
			Left	Parietal cortex (inferior, superior, intra-parietal sulcus)	7/40	−38	−44	34	1	8.49***
			Left	Parietal cortex (inferior, superior, intra-parietal sulcus)	7/40	−28	−44	42	2	8.45***
			Right	Insula	13	30	22	8	270	6.96*
			Left	Parietal cortex (inferior, superior, intra-parietal sulcus)	7/40	−30	−46	42	345	6.88*
			Right	Parietal cortex (inferior)	39/40	36	−52	46	187	6.43*
			Left	Medial frontal gyrus (including anterior cingulate gyrus (dorsal part))	6/8/32	−2	14	52	110	4.86
			Right	Dorsolateral prefrontal cortex	9	48	36	26	38	3.75
			Left	Insula	13	−32	24	2	61	3.71
			Right	Parietal cortex (superior)	7	16	−68	60	47	3.68
Interaction effects										
		Interaction 1: DID-G						n.s.		
		Interaction 2: DID-G						n.s.		
		Interaction 1: DID-S						n.s.		
		Interaction 2: DID-S								
			Right	Insula	13	34	18	4	37	4.17
Interaction 1: controls								
			Left	Putamen	49	−22	14	4	5	5.07**
			Right	Parietal cortex (superior) and praecuneus	7	6	−62	54	202	5.00**
			Right	Medial frontal gyrus (including anterior cingulate gyrus (dorsal part))	6/32	4	12	52	55	4.09
			Right	Parietal cortex (inferior, superior, intra-parietal sulcus)	7/40	44	−46	36	71	3.82
		Interaction 2: controls						n.s.		
Between group	Between-group comparisons									
		DID-G NIS > DID-S NIS						n.s.		
		DID-S NIS > DID-G NIS								
			Left	Ventrolateral prefrontal cortex	44/45	−54	22	20	84	3.69
		DID-G TIS > DID-S TIS						n.s.		
		DID-S TIS > DID-G TIS								
			Right	Praecuneus	7	8	−68	48	52	3.53
			Right	Dorsolateral prefrontal cortex	9	46	24	24	15	3.23
		DID-G NIS > CTRL-HC						n.s.		
		CTRL-HC > DID-G NIS								
			Right	Parietal cortex (inferior, superior, intra-parietal sulcus)	7/40	40	−44	34	10	4.46**
			Right	Praecuneus	7	6	−62	52	41	4.05
			Right	Medial frontal gyrus	6	0	12	52	45	3.30
		DID-G TIS > CTRL-PTSD								
			Left	Anterior cingulate gyrus (dorsal part)	32	0	2	46	14	3.42
			Right	Parietal cortex (inferior, superior, intra-parietal sulcus)	7/40	44	−48	32	9	3.23
		CTRL-PTSD > DID-G TIS						n.s.		
	Interactions between groups									
		Interaction 1: DID-G > DID-S						n.s.		
		Interaction 2: DID-G > DID-S						n.s.		
		Interaction 1: DID-G > controls						n.s.		
		Interaction 2: DID-G > controls								
			Right	Praecuneus	7	4	−62	54	42	3.42
			Right	Medial frontal gyrus (including anterior cingulate gyrus (dorsal part))	6/32	4	12	48	16	3.00

Activated brain regions for main and interaction effects and within- and between-group comparisons for main working memory. Brain regions associated with working memory functioning are listed according to group (DID-G, DID-S and controls) and state (NIS and TIS). The control group consisted of PTSD as the TIS and CTRL-HC as the NIS. Interaction 1 = MWM × DPS = (1, 2, 3 back > 0 back) × (NIS > TIS). Interaction 2 = MWM × DPS = (1, 2, 3 back > 0 back) × (TIS > NIS). n.s. indicates that the value was not significant at exploratory *P* < 0.005 uncorrected or *k* < 9. CTRL-HC, healthy controls; MNI, Montreal Neurological Institute coordinate space; DID-G, diagnosed genuine dissociative identity disorder; NIS, neutral identity state; TIS, trauma-related identity state; DID-S, simulated dissociative identity disorder; PTSD, post-traumatic stress disorder; CTRL-PTSD, PTSD controls; MWM, main working memory; DPS, dissociative personality state; SVC, small volume correction.

* *P* < 0.05 corrected for multiple comparisons for the regions of interest included in the SVC mask.

** *P*-value between 0.05 and <0.1 corrected for multiple comparisons for the regions of interest included in the SVC mask. Results from exploratory analysis at a *P*-value between 0.005 and <0.000 and *k* ≥ 9 uncorrected are unmarked.

*** *P* < 0.05 corrected for multiple comparisons for the whole brain.

**** *P* < 0.05 family-wise error-corrected for multiple comparisons for the regions of interest included in the SVC mask and *P* < 0.05 family-wise error-corrected for multiple comparisons for the whole brain.

##### Within group: interaction effects

Within-group comparisons of identity state-dependent MWM brain activation (see the ‘Interactions within groups’ MWM section in Table 2) showed that the NIS control group (healthy controls) activated the right parietal and medial prefrontal cortex and the left putamen more than the TIS control group (the PTSD group). The TIS of the DID-S group activated the right insula more than the NIS of the DID-S group. There were no significant differences between the brain activation patterns of NIS and TIS in the DID-G group, with regards to MWM.

##### Between-group comparisons

Between-group comparisons of the NIS revealed that MWM-related brain regions showed more activation in the NIS of the DID-S group in the left VLPFC as compared with the NIS of the DID-G group. When compared with the NIS of the DID-G group, the NIS of the control group showed more activation in the right parietal cortex, praecuneus and medial frontal gyrus. Regarding the between-group comparison of the TIS, we found that the TIS of the DID-S group showed increased activation in the right DLPC and praecuneus compared with the TIS of the DID-G group. We also found that the TIS of the DID-G group showed increased activation of the left dorsal anterior cingulate and right parietal cortex as compared with the TIS of the control group.

##### Between group: interaction effects

Between-group comparison of identity state-dependent MWM brain activation (see the ‘Interactions between groups’ MWM section in Table 2) showed that the difference between the TIS and NIS of the DID-G group was larger than the difference between the TIS and NIS of the controls in the right praecuneus and right medial frontal gyrus.

#### Neural correlates of linear (task) load

##### Within group: main effects of task

The brain activation patterns of the main effects for task load were similar to those shown for MWM. All groups showed activation in the PPN, but the extent of the activation was different across identity states and groups (see bottom parts of Fig. 2 and Table 3). Activation of the right insula, right dorsolateral prefrontal cortex and left medial frontal cortex was present in all groups and identity states. The TIS of the DID-G group was the only identity state that did not engage the parietal cortex for task load.

**Table 3:** Group and identity state-dependent neural correlates of linear load

				Linear load		MNI				
			Right/left	Brain region	Brodmann area	*x*	*y*	*z*	*k*	*T*
Within group	Main effects of task	DID-G								
		NIS main effect								
			Right	Parietal cortex (superior)	7	18	−66	58	133	7.41*
			Right	Insula	13	32	20	6	235	6.37*
			Right	Parietal cortex (inferior, superior, intra-parietal sulcus)	7/40	34	−48	36	365	5.72**
			Left	Parietal cortex (inferior, superior, intra-parietal sulcus)	7/40	−32	−46	38	296	5.41**
			Left	Medial frontal gyrus (including anterior cingulate gyrus (dorsal part))	6/32	−8	12	50	108	4.89
			Right	Dorsolateral prefrontal cortex (including frontal pole)	9	46	36	36	91	4.33
			Left	Ventrolateral prefrontal cortex	44	−52	16	26	55	3.97
			Left	Frontal pole	10	−40	50	14	39	3.72
			Left	Insula	13	−34	24	4	23	3.49
		TIS main effect								
			Right	Insula	13	36	24	−6	141	5.22
			Left	Medial frontal gyrus (including anterior cingulate gyrus (dorsal part))	6/32	−10	10	44	73	4.44
			Right	Dorsolateral prefrontal cortex	9	42	38	36	22	3.69
			Right	Dorsolateral prefrontal cortex	9	40	28	34	11	3.47
		DID-S								
		NIS main effect								
			Left	Parietal cortex (inferior)	40	−50	−44	52	60	12.68***
			Right	Parietal cortex (inferior, superior, intra-parietal sulcus)	7/40	38	−44	38	165	9.94***
			Left	Frontal gyrus (middle, superior)	6	−34	−6	62	8	9.05***
			Right	Frontal gyrus (middle, superior)	6	22	6	60	3	8.55***
			Right	Premotor cortex	6	28	−8	56	5	8.37***
			Left	Premotor cortex	6	−44	8	30	2	8.27***
			Left	Parietal cortex (inferior, superior, intra-parietal sulcus)	7/40	−26	−56	40	1	8.24***
			Left	Premotor cortex	6	−26	−2	58	3	8.24***
			Right	Parietal cortex (inferior, superior, intra-parietal sulcus)	7/40	38	−44	38	380	9.94
			Right	Dorsolateral prefrontal cortex (including frontal gyrus (middle))	9/46	50	28	30	479	9.20****
			Left	Parietal cortex (inferior, superior, intra-parietal sulcus)	7	−26	−54	42	481	7.91*
			Left	Dorsolateral prefrontal cortex (including ventrolateral prefrontal cortex)	44/46	−42	10	26	442	7.37*
			Left	Medial frontal gyrus (including anterior cingulate gyrus (dorsal part))	6/32	−4	12	54	245	7.22*
			Right	Insula (including putamen)	13	38	24	−2	273	6.01*
			Left	Frontal pole	10/46	−30	48	24	232	5.78*
			Right	Parietal cortex (superior)	7	12	−76	52	312	5.11**
			Left	Insula	13/47	−30	28	−2	93	4.80
		TIS main effect								
			Left	Parietal cortex (inferior, superior, intra-parietal sulcus)	7/40	−40	−46	34	17	8.90***
			Left	Parietal cortex (inferior)	40	−50	−40	42	13	8.85***
			Left	Parietal cortex (superior)	7	−28	−62	48	8	8.79***
			Right	Parietal cortex (angular)	39	38	−64	50	1	8.42***
			Right	Parietal cortex (angular)	39	36	−66	48	1	8.23***
			Left	Parietal cortex (inferior, superior, intra-parietal sulcus)	7/40	−40	−48	36	474	8.33***
			Right	Parietal cortex (inferior, superior, intra-parietal sulcus)	7/40	38	−46	36	370	8.19****
			Left	Ventrolateral prefrontal cortex	6/44/46	−46	10	28	327	7.38*
			Left	Insula	13/45	−34	18	8	210	6.89*
			Right	Parietal cortex (superior, praecuneus)	7	10	−68	56	412	6.86*
			Right	Insula	13	36	22	4	336	6.59*
			Right	Dorsolateral prefrontal cortex	9	42	40	30	329	5.96*
			Left	Frontal pole	10	−34	50	16	112	5.52*
			Left	Medial frontal gyrus (including anterior cingulate gyrus (dorsal part))	6/8/32	−2	16	54	268	5.31**
			Left	Frontal pole	10	−44	40	24	34	3.95
		Controls								
		Healthy controls main effect	
			Left	Medial frontal gyrus (superior)	8	−4	32	40	19	9.84***
			Right	Medial frontal gyrus (superior)	8	4	22	46	10	8.88***
			Right	Parietal cortex (inferior, superior, intra-parietal sulcus)	7/40	44	−46	40	406	11.04****
			Right	Insula	13	38	26	−2	311	10.21****
			Right	Medial frontal gyrus (including anterior cingulate gyrus (dorsal part))	6/8/32	4	18	46	235	7.30*
			Left	Parietal cortex (inferior, superior, intra-parietal sulcus)	7/40	−28	−58	42	471	7.21*
			Left	Insula	13	−30	22	2	239	7.06*
			Left	Ventrolateral prefrontal cortex	44	−52	18	26	318	6.42*
			Right	Parietal cortex (superior, praecuneus)	7	6	−72	52	428	6.24*
			Right	Dorsolateral prefrontal cortex	9	46	32	32	342	6.05*
			Left	Frontal pole	10	−36	54	20	48	5.04**
			Left	Putamen	49	−22	14	4	10	3.79
		PTSD main effect								
			Left	Parietal cortex (inferior, superior, intra-parietal sulcus)	7/40	−32	−46	42	358	8.76****
			Right	Insula	13/45	30	20	8	261	6.62*
			Left	Medial frontal gyrus (including anterior cingulate gyrus (dorsal part))	6/8/32	−4	14	52	188	6.35*
			Right	Parietal cortex (inferior, superior, intra-parietal sulcus)	7/40	40	−52	48	216	5.57*
			Right	Parietal cortex (superior, praecuneus)	7	18	−72	58	136	4.79
			Left	Insula	13	−30	22	2	98	4.57
			Left	Ventrolateral prefrontal cortex	44	−48	12	16	22	4.47
			Right	Dorsolateral prefrontal cortex (including frontal pole)	9	48	34	28	91	4.41
			Left	Frontal pole	10	−36	52	24	34	3.68
			Left	Ventrolateral prefrontal cortex	9/44	−44	16	24	30	3.26
	Interactions within groups									
		Interaction 3: DID-G						n.s.		
		Interaction 4: DID-G						n.s.		
		Interaction 3: DID-S						n.s.		
		Interaction 4: DID-S						n.s.		
		Interaction 3: controls								
			Right	Praecuneus	7	4	−62	54	119	4.58
			Right	Parietal cortex (inferior, superior, intra-parietal sulcus)	7/40	44	−46	36	61	3.95
		Interaction 4: controls								
			Left	Anterior cingulate gyrus (dorsal part)	32	−2	4	38	20	4.54
Between group	Between groups									
		DID-G NIS > DID-S NIS						n.s.		
		DID-S NIS > DID-G NIS								
			Right	Parietal cortex (inferior, superior, intra-parietal sulcus)	7/40	36	−44	36	18	4.10
			Left	Frontal pole	10	−30	48	24	38	4.06
			Left	Ventrolateral prefrontal cortex	44	−54	20	20	199	4.02
			Right	Medial frontal gyrus (including anterior cingulate gyrus (dorsal part))	6/8/32	4	14	50	89	3.65
			Right	Praecuneus	7	10	−72	46	9	2.96
		DID-G TIS > DID-S TIS						n.s.		
		DID-S TIS > DID-G TIS								
			Left	Ventrolateral prefrontal cortex	44/46	−54	22	24	36	3.62
			Right	Praecuneus	7	8	−72	46	83	3.58
			Left	Parietal cortex (inferior)	7/40	−30	−56	36	15	3.26
			Left	Medial frontal gyrus (including anterior cingulate gyrus (dorsal part))	6/8/32	−6	12	54	23	3.22
			Right	Parietal cortex (inferior, superior, intra-parietal sulcus)	7/40	40	−44	36	12	3.17
			Left	Ventrolateral prefrontal cortex	44	−52	10	22	12	3.16
			Right	Parietal cortex (superior, praecuneus)	7	16	−70	58	18	3.11
		DID-G NIS > CTRL-HC						n.s.		
		CTRL-HC > DID-G NIS								
			Right	Medial frontal gyrus (including anterior cingulate gyrus (dorsal part))	6/8/32	4	16	48	94	3.89
			Right	Parietal cortex (inferior, superior, intra-parietal sulcus)	7/40	40	−44	36	14	3.75
			Right	Praecuneus	7	6	−62	52	27	3.05
		DID-G TIS > CTRL-PTSD						n.s.		
		CTRL-PTSD > DID-G TIS						n.s.		
	Interaction between groups									
		Interaction 3: DID-G > DID-S						n.s.		
		Interaction 4: DID-G > DID-S						n.s.		
		Interaction 3: DID-G > controls						n.s.		
		Interaction 4: DID-G > controls						n.s.		

Activated brain regions for main and interaction effects and within- and between-group comparisons for linear load. Brain regions associated with working memory functioning are listed according to group (DID-G, DID-S and controls) and state (NIS and TIS). The control group consisted of PTSD as the TIS and CTRL-HC as the NIS. Interaction 3 = linear load × DPS = (0 < 1 < 2 < 3 back) × (NIS > TIS). Interaction 4 = linear load × DPS = (0 < 1 < 2 < 3 back) × (TIS > NIS). n.s. indicates that the value was not significant at exploratory *P* < 0.005 uncorrected or *k* < 9. CTRL-HC, healthy controls; MNI, Montreal Neurological Institute coordinate space; DID-G, diagnosed genuine dissociative identity disorder; NIS, neutral identity state; TIS, trauma-related identity state; DID-S, simulated dissociative identity disorder; PTSD, post-traumatic stress disorder; CTRL-PTSD, PTSD controls; DPS, dissociative personality state; SVC, small volume correction.

* *P* < 0.05 corrected for multiple comparisons for the regions of interest included in the SVC mask.

** *P*-value between 0.05 and <0.1 corrected for multiple comparisons for the regions of interest included in the SVC mask. Results from exploratory analysis at a *P*-value between 0.005 and <0.000 and *k* ≥ 9 uncorrected are unmarked.

*** *P* < 0.05 corrected for multiple comparisons for the whole brain.

**** *P* < 0.05 family-wise error-corrected for multiple comparisons for the regions of interest included in the SVC mask and *P* < 0.05 family-wise error-corrected for multiple comparisons for the whole brain.

##### Within group: interaction effects

Within-group comparisons of identity state-dependent linear load brain activation (see the ‘Interactions within groups’ linear load section in Table 3) showed that the NIS control group activated the right parietal cortex and the right praecuneus more than the TIS control group. The TIS control group activated the left dorsal anterior cingulate insula more than the NIS. There were no significant differences in brain activation patterns between the NIS and TIS of the DID-G and DID-S groups.

##### Between-group comparisons

Between-group comparisons of the NIS revealed that linear load-related brain regions showed more activation in DID-S group as compared with the NIS of the DID-G group in the right parietal cortex, medial frontal gyrus and praecuneus, and the left frontal pole and VLPFC. More linear load-dependent brain activation in the NIS of the control group as compared with the NIS of the DID-G group was found in the right parietal cortex, praecuneus and medial frontal gyrus. Regarding the between-group comparison of the TIS of the DID-S group and the DID-G group, we found that the TIS of the DID-S group showed increased activation of the bilateral parietal cortex, left VLPC and medial frontal gyrus, and right praecuneus. Brain activation in the TIS of the DID-G group did not differ from that of the control group.

##### Between group: interaction effects

Between-group comparison of identity state-dependent differences of linear load-related brain activation (see the ‘Interactions between groups’ linear load section in Table 3) showed that there were no significant differences between groups.

## Discussion

NIS- and TIS-dependent behavioural performance and neural activation patterns during a working memory task were investigated for the first time in those with diagnosed DID, and compared with DID simulators and a paired control group consisting of those with PTSD and healthy controls. Our most important finding was that behavioural performance and brain activation patterns related to working memory are dissociative identity state-dependent. A second important finding was that the simulating controls were not able to mimic the behavioural performance and identity state-dependent differences in brain activation observed in those with genuine DID.

### Identity state-dependent working memory performance

We found dissociative identity state-dependent behavioural performance and activation of the PPN. Authentic and simulated TIS had slower reactions to the *n*-back task, with the greatest effect for authentic TIS (Fig. 1(a)). There was a group × identity state interaction (trend significance). This is depicted in Fig. 1(a) as the greater (and in the opposite direction) effects of dissociative identity state in the DID-G and DID-S groups compared with controls. Fig. 2 provides a visual representation of the neurobiological effect and, although the difference between activation patterns for NIS and TIS was not significant, they present a pattern that supports the behavioural data. The pattern shows that the DID-G group's TIS activated the PPN to a lesser extent than the NIS, particularly in the left VLPFC and frontal pole. These findings are in line with the first hypothesis. Similar activation patterns were found for the TIS of the DID-G and PTSD groups for MWM, suggesting similarities in working memory disturbances in the TIS of these groups. The DID-S group showed increased MWM-related brain activation of the right insula in the TIS compared with their simulated NIS, which is the opposite of what we hypothesised. When directly comparing the TIS of the DID-S group with that of the DID-G group, we found increased activation in multiple nodes of the PPN for the DID-S group for both the MWM and linear task load. This latter finding is consistent with our third hypothesis, namely that DID simulators are not able to simulate the working memory-related neural activation patterns of those with diagnosed DID. This coincides with the finding that for linear load, the TIS of the DID-G group did not engage the parietal regions.

Although the reaction time data for both the DID-G and DID-S groups showed similarly slowed reaction times in their TIS, the DID-G group was more likely to make errors of omission on the *n*-back task in their TIS. Trials lacking a response (errors of omission) are censored observations of reaction time and do not contribute to the reaction time analysis. If the increased omission rate was only produced by severely slowed reaction time, then we would also expect more errors of omission in the DID-S group. The absence of omission errors in the DID-S group may be explained by unawareness of having to simulate errors of omission as well as slowed reaction time. This failure in the fidelity of the simulation further supports the third hypothesis. In addition, greater performance accuracy, coupled with similar response speed and increased neural activation during the *n*-back task in the DID-S group compared with the DID-G group, is in line with the first hypothesis that working memory and executive functioning are impaired in dissociative disorders.^3,29,30^ However, our results contrast with studies suggesting enhanced working memory functioning related to dissociation.^8,31,32^

The observed differences in neural activation patterns between authentic NIS and TIS during a working memory task corroborate and extend previous neuroimaging results suggesting differences in brain functioning between these prototypical dissociative identity states.^33^ As hypothesised, TIS showed poorer behavioural performance and impaired working memory functioning compared with NIS, as reflected by less activation in the PPN and lower performance accuracy. We propose two explanations for these findings. First, the behavioural results in TIS might be a result of intrusions, such as dissociative flashbacks, re-experiencing traumatising events, and intruding voices, thoughts, movements, emotions and/or physical sensations, which are key symptoms in dissociative disorders and affect cognitive functioning.^34–36^ It is clinically observed that those in a TIS are preoccupied with traumatic memories, and prone to engage in re-experiencing these.^13,16^ This re-experiencing of trauma in TIS is typically sensorimotor and highly emotional in nature, and associated with reduced mentalising capacity. In contrast to TIS, the DID-G group was better able to perform the *n*-back task in the NIS, ruling out general executive impairments as a trait characteristic. We note that a lack of general executive impairments is consistent with clinical observations. Second, our results could be considered in relation to the retracted field of consciousness in TIS.^13,16^ Reduced PPN activity in a TIS may be related to this retracted field of consciousness and lack of presentation in DID, i.e., that those in a TIS are largely stuck in the past.^13,16^ Poor working memory performance could possibly be regarded as a state feature of TIS, suggesting a preoccupation with trauma-related associations and possibly more internally focused attention.

### Clinical implications

Our findings have importance for individuals with DID, and more widely for individuals with PTSD, and possibly for the general trauma field. These wider implications arise because in TIS, but not NIS, patients with DID are prone to relive traumatic memories; thus, in DID, the identity state-dependent differences in working memory performance are likely linked to reliving traumatic memories. In part, this is because NIS is an identity state in which patients attempt to distance themselves from a traumatic past, thereby leaving the TIS fixated in that past. The working memory capacity of NIS is higher than that of TIS, and better working memory capacity is associated with the ability to inhibit irrelevant or disturbing cues and more effective suppression of negative, personally relevant thoughts in suppression tasks.^37^ In DID, emotion regulation highly depends on working memory,^38^ and TIS is thought to be a hyperaroused identity state undermodulating emotion.^14^ Therefore, improvement of working memory is believed to enhance emotion regulation,^39^ since training emotional working memory enhances the efficiency of the PPN,^40^ as well as cognitive and emotional performance in PTSD.^41^ In DID treatment, clinicians aim to generate a wider field of consciousness in the TIS and promote working memory function. This is achieved by improving communication between, and cooperation of, TIS and NIS during phase-oriented treatment of DID.^13^ TIS can further be assisted by using eye movement desensitisation and reprocessing (EMDR),^42^ as previous neuroimaging studies have reported increased activation in prefrontal brain regions known to be involved in cognitive control after the completion of successful EMDR treatments in PTSD.^43,44^

Although the PTSD group was not the main group under investigation, the results presented in this paper are important for the treatment of individuals with PTSD, and possibly for treatment of traumatised individuals in general. Results from both the behavioural data (Fig. 1(a)) and neural activation data (Tables 2 and 3, ‘PTSD main effect’ and ‘Interaction: Controls’) shows that working memory capacity is higher in healthy controls than in individuals with PTSD. Therefore, based on our findings and as described above, we recommend promoting working memory function during treatment not only in the TIS of those with DID, but in PTSD as well.

### Study considerations regarding the trauma and fantasy models

According to the trauma model,^15,19,45^ DID is the most severe of trauma-related psychiatric disorders, being on the far end of PTSD. This model postulates that the experience of early childhood traumatisation and high levels of stress are related to cognitive deficits. However, the alternative fantasy or sociocognitive (non-trauma-related) model states that DID can easily be simulated in motivated individuals. The most replicated difference between DID and simulating controls is a deficit in cognitive processing in DID for memory and reaction times in general,^6,46,47^ but identity state-dependent neural correlates of these differences remain unexplored. It is therefore important to investigate identity state-dependent working memory functioning in DID compared with working memory functioning in simulated DID, as well as in healthy controls and individuals with PTSD.

According to the trauma model, individuals with DID would be expected to resemble individuals with PTSD and to differ from DID simulators. Specifically, it can be hypothesised that working memory abnormalities are mainly present in TIS, whereas NIS displays a higher level of integrative capacity and mental efficiency,^16^ and would therefore be more similar to healthy controls. The expectation that working memory is most abnormal in the TIS in genuine DID is based on the premise that emotion regulation highly depends on working memory,^38^ and that TIS is a hyperaroused identity state that undermodulates emotion.^14^ According to the trauma model, but not the fantasy model, DID-S controls are expected to be unable to mimic these abnormalities.

### Trauma versus fantasy model of DID

According to the trauma model, behavioural and brain activation differences between NIS and TIS were expected. More specifically, neural activation patterns and behavioural performance in the TIS of the DID-G group would be negatively affected. Our study confirmed these ideas. The overall picture of neural activation patterns, as shown in Fig. 2, is in line with the behavioural data, our first hypothesis and the trauma model for DID. It may also be argued that both NIS and TIS were affected in the DID-G group, considering previous studies showed impaired working memory related to dissociation.^3,29,30^ Impaired functioning in both NIS and TIS is also a general hypothesis of the theory of structural dissociation of the personality.^13,16^ This theory additionally hypothesises that the integrative capacity of individuals with DID is lower than in mentally healthy individuals, particularly in stressful situations. Furthermore, studies have reported trauma-related hypoactivation of regions involved in attention and working memory in PTSD.^4^ Hence, although we did not find support for the hypothesis of equal or enhanced working memory performance and increased neural PPN activation in the NIS of the DID-G group as compared with healthy controls,^8^ our findings support the theory of structural dissociation of the personality.

The fantasy model of DID states that DID can easily be simulated in motivated individuals.^19,45^ Opposing the fantasy model for DID, we expected differences in behavioural performance and neural activation patters between the DID-G and DID-S groups. Regarding behavioural performance, the DID-S group were not able to perfectly replicate the features of reaction time performance in the DID-G group, and discrepancies in the rate of omission errors were even more substantially different. Increased brain activation was found for the within-group comparisons, where the simulated TIS of the DID-S group showed increased brain activation as compared with its simulated NIS. With regards to the between-group comparison, we found that the TIS and NIS of the DID-S group showed increased activation in working memory-related brain areas compared with the TIS and NIS of the DID-G group. The inability to enact behavioural and neural responses found in our study is in line with previous studies showing deficits in cognitive processing in DID compared with simulating controls,^6,46,47^ and studies showing differences in neural activation patterns between diagnosed and simulated DID.^14,15,48–50^ Taken together, these studies and the present study suggest that mentally healthy controls are unable to simulate crucial patterns found in those with diagnosed DID, which contrasts with a core hypothesis of the fantasy model of DID. Based on this evidence, we propose that future research does not need to include a DID-S group.^5^

### Limitations

Some limitations of the present study should be noted. The current study includes a modest sample size of 14 participants of DID and 32 control participants, partly because of the inherent challenge of investigating effects between identity states: the study required multiple identity states and the ability to predictably switch between them. For the brain imaging data, group differences for direct comparisons were mainly found when applying less stringent thresholds, but our study is the only study to date investigating identity state-dependent behavioural performance and brain activation of working memory performance, as well as controlling for motivated role-playing. Results of this study could therefore still provide an important contribution to the literature. Only female patients and controls were studied, so that our findings cannot be extended to DID populations in general. However, a single-gender sample has the advantage of excluding gender differences known to be present for brain activity during working memory performance.^51^ Finally, psychotropic medication intake differed between patients and controls, which could have influenced our results, but no cumulative effect is expected because type and dose varied within the DID-G group. It is also unlikely that medication use influenced identity state differences in the DID-G group, since both NIS and TIS would be affected. Despite these limitations, our study is currently the largest study investigating dissociative identity state-dependent brain activation of working memory functioning.

## Data Availability

The data that support the findings of this study are available from the corresponding author, A.A.T.S.R., upon reasonable request.

## Supplementary material

For supplementary material accompanying this paper visit http://doi.org/10.1192/bjo.2022.22. click here to view supplementary material
