# Supplementary material for: Dissociative identity state-dependent working memory in dissociative identity disorder: a controlled functional magnetic resonance imaging study
Source: BJPsych Open. 2022 Apr 11;8(3):e82. doi: 10.1192/bjo.2022.22 (PMC9059616; doi:10.1192/bjo.2022.22)
Supplement: Supplementary file 1 [file bjosup.zip › S2056472422000229sup001.docx]

**SUPPLEMENTARY MATERIALS**

**FOR THE MANUSCRIPT:**

**Dissociative identity state-dependent working memory in dissociative identity disorder:**

**a controlled fMRI study**

Eline M. Vissia*, Andrew J. Lawrence*, Sima Chalavi, Mechteld E. Giesen,

Nel Draijer, Ellert R.S. Nijenhuis,

André Aleman, Dick J. Veltman, Antje A.T.S. Reinders

**SUPPLEMENTARY MATERIAL 1**

**PARTICIPANTS**

Individuals with PTSD with a history of interpersonal traumatizing events and individuals with DID were recruited via mental healthcare institutions and internet advertisements. Symptom severity in the individuals with PTSD was assessed using the Clinician Administered PTSD Scale (CAPS) interview (1) performed by researchers M.G and E.V. The diagnosis of DID was assessed by one of two DID experts (N.D. or E.N.) on the basis of the Structural Clinical Interview for DSM-IV Dissociative Disorders (SCID-D (2), Dutch translation (3,4)). As previously specified (5,6) the therapists of the patients with DID reported the following co-morbid disorders, based on clinical DSM-IV classification: chronic PTSD (n=3), PTSD (n=2), somatoform disorder (n=2), recurrent major depression (n=4), dysthymic disorder (n=1), trauma-related specific phobias (n=2), personality disorder not otherwise specified (n=1), mixed personality disorders (n=2), borderline personality disorder symptoms (n=3), dependent personality disorder symptoms (n=1), histrionic personality disorder symptoms (n=1) eating disorder (n=2), sleeping disorder (n=2) and catalepsy (n=1). Exclusion criteria were age outside 18-65, pregnancy, systemic/neurological illness, claustrophobia, presence of metal implants and alcohol/drug abuse.

DID simulating healthy controls (DID-S) were recruited from acting schools, through advertisements on the website [www.theaternetwerk.nl](http://www.theaternetwerk.nl/) and in magazines and newspapers. Instructions for simulation practice and performance have been described in detail previously (7,8)**.** All the actors had at least two years experience with acting. HC were recruited through advertisements in local newspapers. Additional exclusion criteria for HC and DID-S were the presence of psychological and somatoform dissociative symptoms, as determined with the Dissociative Experiences Scale (DES (9)) and Somatoform Dissociation Questionnaire (SDQ-20 (10)) and a high score on the Traumatic Experience Checklist (TEC (11)) or mental illness in the past or at present. Healthy (simulating) controls were required to score below a cut-off point of 25 on the DES and 29 on the SDQ-20 as part of inclusion criteria.

Demographic data were analysed with R employing permutation tests for categorical data, one-way ANOVA for continuous approximately normal variables, and nonparametric Kruskal-Wallis tests otherwise (see Table S1). Table S1 presents the demographics of the four groups: DID-G, DID-S, HC, and PTSD. There were no significant differences in age or level of education. Groups differed significantly on psychoform (DES) and somatoform (SDQ-20) dissociative symptoms, general levels of anxiety (STAI-T) and reported adverse experiences (TEC). As expected, post-hoc tests revealed no significant differences between the two non-psychiatric HC and DID-S groups on symptom measures, and for all four measures the psychiatric (DID-G and PTSD) groups had elevated scores relative to HC and DID-S. For the DES, SDQ-20, and TEC the DID-G group had significantly more severe dissociative symptoms and reported more adverse life events than the PTSD group. However, this direction of effect was reversed for the STAI-T anxiety measure (albeit non-significantly; p_adj_=0.42), which fits the fact that DID-G completed the questionnaires as NIS who typically avoid affect.

**N-BACK TASK-DESCRIPTION**

A visual letter n-back task was used to investigate WM-functioning. Participants viewed single capital letters projected onto a screen one after another and were required to press a response key when the projected letter was the same as the last shown letter (1-back), the same as the letter preceding the last shown letter (2-back), or the same as the letter preceding the last two shown letters (3-back). They were also instructed to press a response key when the letter 'x' appeared on the screen (baseline or 0-back). A condition-specific instruction was shown each time a new condition started. Each condition consisted of 14 stimuli with three targets. Each condition was presented three times, in a pseudo-randomized order, resulting in a total of 12 blocks of each 14 stimuli, giving a total of 9 targets per difficulty. Each stimulus was presented for one second with an inter-stimulus interval of one second and the total duration of the task was approximately 7 minutes. Behavioural data was obtained to allow for the assessment of reaction times and errors of omission. We used different playlists (12,13): two per session (3 sessions: 2x practice, 1x fMRI). Per session, the two different versions available were counterbalanced, so half of the time DID-G NIS/DID-S NIS got the first version and DID-G TIS/DID-S TIS the second version and half of the time this was reversed. Participants practiced the n-back task until the investigator was confident that the participants fully understood the instructions. The DID-S were instructed to simulate DID during this part of the research. Both DID-G and DID-S went through this session twice, once as NIS and once as TIS. During the whole second part it was verified by one of the researchers (M.G. or E.V.) that DID-G and DID-S were present as the authentic and simulated NIS and TIS, respectively, at the requested instances. Of note, some TIS engage in this context in a mammalian defence pattern that involves hypoarousal, anaesthesia, as well as emotional and physical numbing. These TIS were not included in the present study.

**BRAIN IMAGING ACQUISITION AND PREPROCESSING DETAILS**

***Image acquisition***

Brain imaging was conducted on a 3T Philips MRI scanner at the Neuroimaging Centre of the UMCG and at AMC. Both centres used the manufacturer’s standard 8-channel head coil. T2*-weighted echo-planar images (EPI) were acquired with the following acquisition parameters: repetition time (TR) = 1700 ms, echo time (TE) = 30 ms, flip angle: 74°, reconstruction matrix: 64x64, slice thickness 2.4 mm, 1mm gap, descending, 32 slices. The first three EPI volumes in each session were discarded post-hoc to allow for magnetic equilibrium effects. In addition, a T1-weighted MR image (14) (voxel size: 1x1x1mm, TR = 9.95ms, TE = 5.6ms, 160 slices) was acquired for co-registration and spatial normalization of the T2*-weighted images. DID-G, DID-S and PTSD and HC participants were scanned in an interleaved order within a relatively short time interval. The samples were distributed over the two centres (8 DID, 9 DID-S, 9 PTSD, and 9 HC were scanned at the UMCG).

***Image preprocessing***

Image processing steps were carried out using the following software packages: FMRIB Software Library (FSL (15), http://fsl.fmrib.ox.ac.uk, v5.0.10), Statistical Parametric Mapping (SPM (16), v12, www.fil.ion.ucl.ac.uk/spm/) and Advanced Normalisation Tools (ANTS, http://stnava.github.io/ANTs/, 2.2.0). Due to a technical problem in functional MRI acquisition the first step of image processing was to apply a denoising script based on FSL MELODIC (17). After this treatment, functional images were realigned to adjust for head motion (FSL mcflirt) and voxel intensities corrected for slice timing (FSL slicetimer). ICA-AROMA ((18,19) v0.4.3, htTIS://github.com/maartenmennes/ICA-AROMA) was then used to identify and filter motion related signal components. A single concatenated spatial transformation was applied to correct for EPI distortion and move to a standard space based on the T1w anatomical scan. This was composed of three registrations as follows: First, an affine boundary based registration (20), was conducted between the mean EPI and the T1w image to align these images (FSL flirt). Second, these results were used to initialise a non-linear unwarping of the mean EPI. This step used an intensity-inverted T1w image and restricted deformations to the phase encode direction affected by EPI distortions (21). Third, each subject’s T1w anatomical image was nonlinearly registered to MNI152 2mm space (ANTS ANTSregistrationSyN.sh). The final registration was a composition of steps 1-3 to unwarp the fMRI images and bring them into standard space. Before statistical analysis an 8 mm full-width-half-maximum Gaussian spatial smoothing was applied. The non-linear unwarping of the functional images was required to correct for susceptibility-induced geometric distortions and thus obtain a good registration to T1w data in the absence of field map data, or images with orthogonal phase-encoding directions (see Wang and colleagues (21)).

**MASK**

We created a mask (see Supplementary materials 1) to be able to apply multiple comparison correction for a smaller volume of specific interest. This mask was created based on the meta-analysis from Owen and colleagues (22) who reported six regions of interest (ROI) related to neural activity underlying the n-back, which were defined *a priori*. These ROIs were all part of the PPN and related to WM: dorsal cingulate/medial premotor (SMA) (BA 32, BA 6); dorsolateral prefrontal (BA 46, BA 9); ventrolateral prefrontal (BA 44); frontal pole (bilateral: BA 10); medial posterior parietal (BA 7); and inferior parietal lobule (bilateral: BA 40). ROIs were constructed using a mask with a 10mm spherical radius around coordinates specified in the meta-analysis (22) and collated in one single mask that was applied to the data using a threshold of p<0.005 uncorrected.

**REFERENCES**

1. Blake DD, Weathers FW, Nagy LM, Kaloupek DG, Gusman FD, Charney DS, et al. The development of a Clinician-Administered PTSD Scale. Journal of traumatic stress. 1995 Jan;8(1):75–90.

2. Steinberg M. Structured clinical interview for DSM-IV dissociative disorders (SCID-D). Washington DC: American Psychiatric Press; 1993.

3. Boon S, Draijer N. Multiple Personality Disorder in The Netherlands: A Clinical Investigation of 71 Patients. Am J Psychiatry. 1993;150(3):489–94.

4. Steinberg M, Boon S, Draijer N. Gestructureerd klinisch interview voor de vaststelling van DSM-IV dissociatieve stoornissen (SCID-D). Lisse: Swets & Zeitlinger; 1994.

5. Reinders AATS, Marquand AF, Schlumpf YR, Chalavi S, Vissia EM, Nijenhuis ERS, et al. Aiding the diagnosis of dissociative identity disorder: pattern recognition study of brain biomarkers. British Journal of Psychiatry [Internet]. 2019 Sep 7 [cited 2019 Oct 25];215(3):536–44. Available from: http://www.ncbi.nlm.nih.gov/pubmed/30523772

6. Reinders AATS, Chalavi S, Schlumpf YR, Vissia EM, Nijenhuis ERS, Jäncke L, et al. Neurodevelopmental origins of abnormal cortical morphology in dissociative identity disorder. Acta Psychiatrica Scandinavica [Internet]. 2018 Feb [cited 2018 Jul 9];137(2):157–70. Available from: http://www.ncbi.nlm.nih.gov/pubmed/29282709

7. Vissia EM, Giesen ME, Chalavi S, Nijenhuis ERS, Draijer N, Brand BL, et al. Is it Trauma- or Fantasy-based? Comparing dissociative identity disorder, post-traumatic stress disorder, simulators, and controls. Acta Psychiatrica Scandinavica. 2016;134(2):111–28.

8. Vissia EM, Giesen ME, Chalavi S, Nijenhuis ERS, Draijer N, Brand BL, et al. Supplementary material for the manuscript: Is it Trauma or Fantasy-based? Comparing Dissociative Identity Disorder, Posttraumatic Stress Disorder, Simulators, and Controls. Acta Psychiatrica Scandinavica [Internet]. 2016 [cited 2020 Jan 21];134(2):1–27. Available from: https://onlinelibrary.wiley.com/action/downloadSupplement?doi=10.1111%2Facps.12590&file=acps12590-sup-0001-SupInfo.pdf

9. Bernstein EM, Putnam FW. Development, reliability, and validity of a dissociation scale. Journal of Nervous and Mental Disease,. 1986;174(12):727–35.

10. Nijenhuis ER, Spinhoven P, van Dyck R, van der Hart O, Vanderlinden J. The development and psychometric characteristics of the Somatoform Dissociation Questionnaire (SDQ-20). The Journal of Nervous and Mental Disease. 1996;184(11):688–94.

11. Nijenhuis ERS, van der Hart O, Kruger K. The psychometric characteristics of the traumatic experiences checklist (TEC): First findings among psychiatric outpatients. Clinical Psychology and Psychotherapy. 2002;9(3):200–10.

12. Elzinga BM, Ardon AM, Heijnis MK, De Ruiter MB, Van Dyck R, Veltman DJ. Neural correlates of enhanced working-memory performance in dissociative disorder: A functional MRI study. Psychological Medicine. 2007 Feb;37(2):235–45.

13. Goozee R, Reinders AATS, Handley R, Marques T, Taylor H, O’Daly O, et al. Effects of aripiprazole and haloperidol on neural activation during the n-back in healthy individuals: A functional MRI study. Schizophrenia Research. 2016 Jun 1;173(3):174–81.

14. Chalavi S, Simmons A, Dijkstra H, Barker GJ, Reinders AS. Quantitative and qualitative assessment of structural magnetic resonance imaging data in a two-center study. BMC Medical Imaging. 2012;12.

15. Jenkinson M, Beckmann CF, Behrens TEJ, Woolrich MW, Smith SM. FSL. NeuroImage. 2012 Aug 15;62(2):782–90.

16. Friston KJ, Jezzard P, Turner R. Analysis of Functional MRI Time-Series. Vol. 1, Human Brain Mapping. 1994.

17. Beckmann CF, Smith SM. Probabilistic Independent Component Analysis for Functional Magnetic Resonance Imaging. IEEE Transactions on Medical Imaging. 2004 Feb;23(2):137–52.

18. Pruim RHR, Mennes M, van Rooij D, Llera A, Buitelaar JK, Beckmann CF. ICA-AROMA: A robust ICA-based strategy for removing motion artifacts from fMRI data. NeuroImage. 2015 May 15;112:267–77.

19. Pruim RHR, Mennes M, Buitelaar JK, Beckmann CF. Evaluation of ICA-AROMA and alternative strategies for motion artifact removal in resting state fMRI. NeuroImage. 2015 May 15;112:278–87.

20. Greve DN, Fischl B. Accurate and robust brain image alignment using boundary-based registration. NeuroImage. 2009 Oct;48(1):63–72.

21. Wang S, Peterson DJ, Gatenby JC, Li W, Grabowski TJ, Madhyastha TM. Evaluation of Field Map and Nonlinear Registration Methods for Correction of Susceptibility Artifacts in Diffusion MRI. Frontiers in Neuroinformatics. 2017 Feb 21;11:17.

22. Owen AM, McMillan KM, Laird AR, Bullmore E. N-back working memory paradigm: A meta-analysis of normative functional neuroimaging studies. Human Brain Mapping. 2005 May;25(1):46–59.

# 23. Piepho HP. An Algorithm for a Letter-Based Representation of All-Pairwise Comparisons.

# 2004;13(2):456-466.
