# Supplementary material for: Dissociative identity state-dependent working memory in dissociative identity disorder: a controlled functional magnetic resonance imaging study
Source: BJPsych Open. 2022 Apr 11;8(3):e82. doi: 10.1192/bjo.2022.22 (PMC9059616; doi:10.1192/bjo.2022.22)
Supplement: Supplementary file 1 [file bjosup.zip › S2056472422000229sup002.docx]

**SUPPLEMENTARY TABLE**

**Table S1: Demographic and clinical characteristics of the participants**

The results presented in this table have been published before as part of the Dutch study only and combined with the Swiss dataset and differ slightly due to different numbers of participants included in the analyses. Of note, as mentioned in the Methods section of the main manuscript data has been analyzed with the statistical package “R” instead of SPSS leading to a different presentation of results. Table S1 presents descriptives by study group alongside omnibus tests for the effect of group. Superscript lowercase letters (a,b,c) indicate the results of post-hoc tests conducted wherever the omnibus effect of group was significant. The letters are a compact letter where groups which do not share a letter significantly differ from each other (p<0.05) in multiplicity adjusted post hoc tests, i.e., superscript letters are a compact letter display giving results of post-hoc pairwise tests between groups (23). Participants were genuine dissociative identity disorder (DID-G), DID simulating healthy controls (DID-S), post- traumatic stress disorder (PTSD) and their paired Healthy Controls (HC). Note that PTSD and HC subjects make up the paired non-simulating control group. Age is treated as normally distributed, ‘Mean (S.D.)’ descriptives are provided with the result of an one-way ANOVA. Years in full time education is treated as categorical with ‘frequency (percentage%)’ descriptives provided alongside a permutation test. Remaining variables were treated as non-normal continuous: ‘Median [Lower Quartile, Upper Quartile]’ descriptives are provided alongside a Kruskall-Wallis omnibus test. Post-hoc tests for questionnaires were Dunn tests with Holm-Bonferroni adjustment for multiple comparisons.

|  | **DID-G** | **DID-S** | **PTSD** | **HC** | **Omnibus** |
| --- | --- | --- | --- | --- | --- |
|  | ***N=14*** | ***N=16*** | ***N=16*** | ***N=16*** | **test** |
| Age (years) | 44.8 (10.3) | 39.7 (13.0) | 42.1 (12.7) | 43.9 (11.0) | 0.688 |
| Education (Years FT) |  |  |  |  | 0.109 |
| <15 | 2 (15.4%) | 3 (20.0%) | 5 (38.5%) | 0 (0.00%) |  |
| 15 | 7 (53.8%) | 5 (33.3%) | 3 (23.1%) | 9 (75.0%) |  |
| 16 | 4 (30.8%) | 7 (46.7%) | 5 (38.5%) | 3 (25.0%) |  |
| DES | 53.2^c^ [43.7;67.7] | 4.64^a^ [3.04;5.89] | 18.9^b^ [11.8;34.7] | 4.46^a^ [3.57;7.59] | <0.001 |
| SDQ-20 | 52.0^c^ [43.2;72.8] | 21.0^a^ [20.0;23.0] | 29.5^b^ [24.8;34.8] | 20.5^a^ [20.0;24.0] | <0.001 |
| STAI-T | 51.0^b^ [42.8;59.2] | 33.0^a^ [30.5;41.0] | 56.0^b^ [51.2;63.5] | 34.0^a^ [29.8;40.0] | <0.001 |
| TEC | 18.0^c^ [16.0;18.8] | 2.00^a^ [0.00;3.50] | 11.0^b^ [9.75;14.2] | 2.00^a^ [0.00;3.00] | <0.001 |

**Abbreviations**:

DID-G= diagnosed genuine dissociative identity disorder

DID-S= simulated dissociative identity disorder

PTSD= posttraumatic stress disorder

HC= healthy controls

DES= Dissociative Experiences Scale

SDQ-20= Somatoform Dissociation Questionnaire

STAI-T= State-Trait Anxiety Inventory-Trait

TEC= Traumatic Experience Checklist

^a^ = denotes groups which do not significantly differ (p>0.05) in post-hoc pairwise comparisons from other groups marked by a superscript a

^b^ = denotes groups which do not significantly differ (p>0.05) in post-hoc pairwise comparisons from other groups marked by a superscript b

^c^ = denotes groups which do not significantly differ (p>0.05) in post-hoc pairwise comparisons from other groups marked by a superscript c
